# Recent Updates on Terpenoids and Other Bioactive Constituents of Marine Sponges

**DOI:** 10.3390/molecules30051112

**Published:** 2025-02-28

**Authors:** Maggie J. F. Raymond, Harinantenaina L. Rakotondraibe

**Affiliations:** Division of Medicinal Chemistry and Pharmacognosy, College of Pharmacy, The Ohio State University, Columbus, OH 43210, USA; raymond.217@buckeyemail.osu.edu

**Keywords:** natural product, bioactivity, marine sponge, terpenoid, alkaloid, peptide

## Abstract

Marine sponges are a promising source of bioactive secondary metabolites, contributing hundreds of novel compounds per year to natural product research, each with diverse chemical and biological properties. We have chosen to highlight marine natural products that exhibited unique structural features and/or significant bioactivity. The most common report of pharmacological significance was cytotoxicity, with antimicrobial and enzyme inhibition activities following, and mentions of other attributes, such as anti-inflammation, neuroprotection, and anti-biofilm. This review describes newly isolated constituents from sponges between 2020 and 2023 alongside their relevant pharmacological activity. The isolation, structures, and biological properties of 218 unique secondary metabolites from various chemical families, including terpenoids, peptides, and alkaloids from marine sponges, are covered.

## 1. Introduction

**Figure 1:**
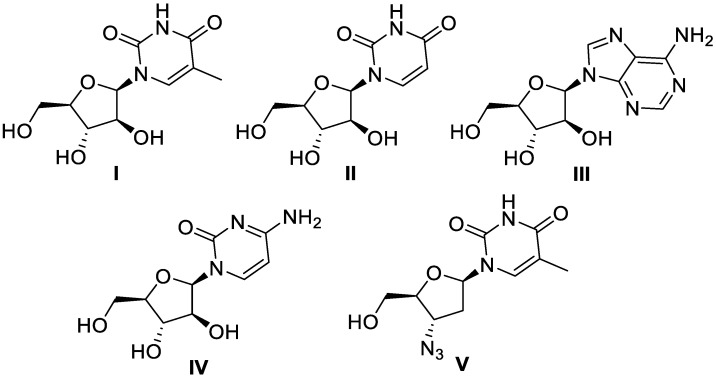
Structures of spongothymidine (**I**), spongouridine (**II**), Ara-A (**III**), Ara-C (**IV**), and AZT (**V**).

Marine organisms have interested scientists working on drug discovery since the 1950s, when Bergmann and Feeney isolated spongothymidine and spongouridine (**I** and **II**, respectively, Figure 1) from the Caribbean sponge *Tethya crypta* [1]. Due to its arabinose sugar moiety and potential for anti-viral and anti-cancer properties, these two compounds altered the perceived notion that nucleosides required a ribose or deoxyribose sugar to display biological activity. The discovery of these nucleosides served as a scaffold for a multitude of synthetic marine-derived drugs, including the anti-viral adenine arabinoside (Ara-A) (**III**, Figure 1), an anti-cancer agent marketed as cytosine arabinoside (Ara-C) (**IV**, Figure 1) for the treatment of leukemia and lymphoma, and azidothymidine (AZT) (**V**, Figure 1), approved in the late 1980s as the first anti-retroviral drug to treat and prevent HIV [2]. Subsequently, the vast biodiversity of marine sponges has proven to be a rich source of remarkable compounds with potent biological activity, ranging from cytotoxic to neuroprotective properties. With more than 6000 known compounds and hundreds of novel metabolites being reported every year, sponges offer promising candidates for pharmacological activity and natural product drug discovery [3]. This review discusses unprecedented metabolites isolated from sponges in a marine environment published within the last three years (2020–2023), which include unique structures compared to bioactive products from other natural sources [4]. As a previous review by Hong et al. covered novel natural products discovered from marine sponges during 2009–2018, we have chosen to highlight the most recent advances and updates [5]. The spike in articles published in 2021 on sponge isolation efforts is a major contribution to the purpose of this discussion. Although many reviews have been published to date on terpenoids from sponges, the current review focuses primarily on the isolation efforts for all types of secondary metabolites [6]. To note, we are only reporting compounds isolated from sponge material and not directly derived from symbionts or microorganisms that co-exist with the invertebrates.

The most abundant classes of chemical constituents from sponges, whose activity and prospective scaffolds are subsequently discussed, include terpenes, alkaloids, and peptides. Terpenoids (isoprenoids) are one of the most abundant groups of natural products derived from sponges and, therefore, are the top focus of this review. Reported bioactive organic small molecules that have been isolated from sponges demonstrated the marine environment’s potential for lead discovery and the development of a variety of new therapeutic agents. Biological activity reported from the subsequent novel compounds covers cytotoxicity, antimicrobial/antibacterial, anti-proliferative, anti-protozoal, anti-tubercular, antimalarial, anthelmintic, anti-viral, anti-prion, anti-biofilm, anti-inflammatory, anti-depressant, neuroprotective, cardioprotective, and enzyme inhibitory effects against chymotrypsin or acetylcholinesterase.

## 2. Terpenoids and Steroids

Terpenes are a class of organic compounds derived from the isoprene precursor scaffolds IPP and DMAPP produced by the mevalonate (MVA) pathway using acetyl-CoA as a substrate or the 2-*C*-methyl-D-erythritol-4-phosphate (MEP) pathway (also called DXP or non-mevalonate pathway) using glyceraldehylde-3-phosphate (GAP) and pyruvate. The structures of terpenoids are generally composed of the head-to-tail conjugation of isoprene units, and their classification is based on the number of fused isoprene units and then individualized by the cyclization and rearrangement of the carbon skeleton followed by functional modifications. Terpenoids (hemi-, mono-, sesqui-, di-, sester-, tri-, and tetra-terpenes) have been known for their diverse range of biological activities supporting their role in traditional plant medicines and dietary supplements [7]. Other types of terpenoids include norterpenoids, which are defined as terpenes that have lost a methyl group, and meroterpenoids, which are hybrid secondary metabolites that are partially derived from terpene biosynthesis but are caused by mixed biosynthetic pathways. Both norterpenes and meroterpenes have been found in sponges and demonstrate unique structures compared to standard terpenoids.

### 2.1. Hemiterpenoids, Monoterpenoids, Tetraterpenoids, and Polyterpenoids

Hemiterpenoids are five carbon-skeleton terpenoids consisting of one isoprene unit. Due to its simplicity, there have not been any reports of hemiterpenoids isolated from marine sponges. Similarly, monoterpenoids comprise two isoprene units (10 carbon-skeleton terpenoids) and are subdivided into acyclic, monocyclic, bicyclic, or irregular types. Although metabolites from this subclass are well-known from sponges, due to limited structural complexity, no novel monoterpenoids isolated from sponges were reported between 2020 and 2023. Tetraterpenoids consist of eight isoprene units (40 carbons), while polyterpenoids are polymeric terpenoid hydrocarbons with more than 40 carbons. The most common tetraterpenoids are fat-soluble pigments known as carotenoids. The polyterpenoid class includes rubbers or high-molecular-weight polymers comprised of *cis*-isoprene units. There were no reports of novel tetraterpenoids or polyterpenoids isolated from sponges between 2020 and 2023; however, tetraterpenoids have been previously reported from sponges, such as *Phorbas gukulensis* [8].

### 2.2. Sesquiterpenoids

Sesquiterpenoids are derived from three isoprene units (15 carbons) and exist with many frameworks, including linear, monocyclic, bicyclic, or tricyclic hydrocarbons.

**Figure 2:**
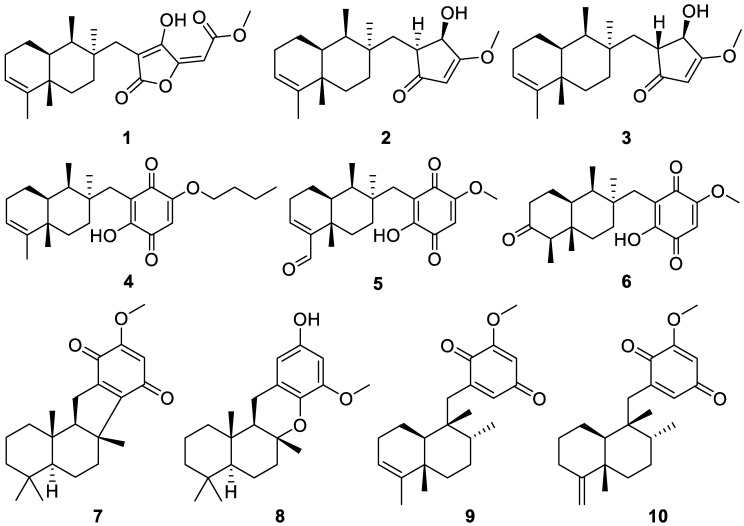
Structures of pseudoceranoids A–J (**1**–**10**).

Investigation of an ethyl acetate fraction of the methanol (MeOH) extract of the sponge *Pseudoceratina purpurea*, collected in the South China Sea, led to the isolation of ten new sesquiterpenes identified as pseudoceranoids A–J (**1**–**10**, Figure 2). Pseudoceranoid A (**1**) is a rare merosesquiterpene crotonolactone derivative with a 4,9-friedodrimane-type core, while the other isolated sesquiterpenes included 4,9-friedodrimane-type (**2**–**6**, **9** and **10**) and drimane-type derivatives (**7** and **8**). Pseudoceranoid D (**4**) showed cytotoxic activity against the following cell lines: human lung carcinoma (H69AR), human leukemia (K562), and human breast cancer (MDA-MB-231), with IC_50_ values of 7.74, 3.01, and 9.82 μM, respectively. Pseudoceranoid E (**5**) was also found to be cytotoxic against the H69AR cell line, with an IC_50_ value of 2.85 μM. Pseudoceranoid F (**6)** displayed cytotoxic activity, with an IC_50_ of 16.14 μM against K562 cells, while Pseudoceranoid H (**8**) exhibited moderate activity against the MDA-MB-231 cell line, with an IC_50_ of 14.01 μM [9].

**Figure 3:**
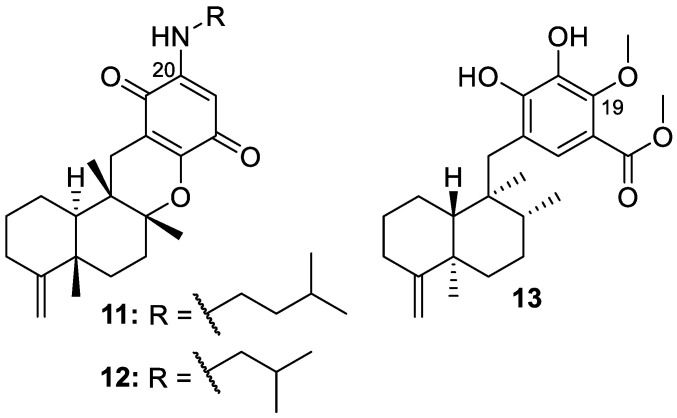
Structures of 20-demethoxy-20-isopentylaminodactyloquinone D (**11**), 20-demethoxy-20- isobutylaminodactyloquinone D (**12**), and 19-methoxy-dictyoceratin-A (**13**).

Isolation efforts on air-dried material of the marine sponge *Dactylospongia elegans* from the South China Sea yielded three new sesquiterpene quinones identified as 20-demethoxy-20-isopentylaminodactyloquinone D (**11**), 20-demethoxy-20-isobutylaminodactyloquinone D (**12**), and 19-methoxy-dictyoceratin-A (**13**) (Figure 3). Of these new isolates, 19-methoxy-dictyoceratin-A (**13**) displayed weak cytotoxicity against the tested cell lines of human prostate cancer (DU145), human pancreatic cancer (SW1990), human liver cancer (Huh7), and human pancreatic carcinoma (PANC-1), with IC_50_ values ranging from 17.4 to 37.8 μM [10].

**Figure 4:**
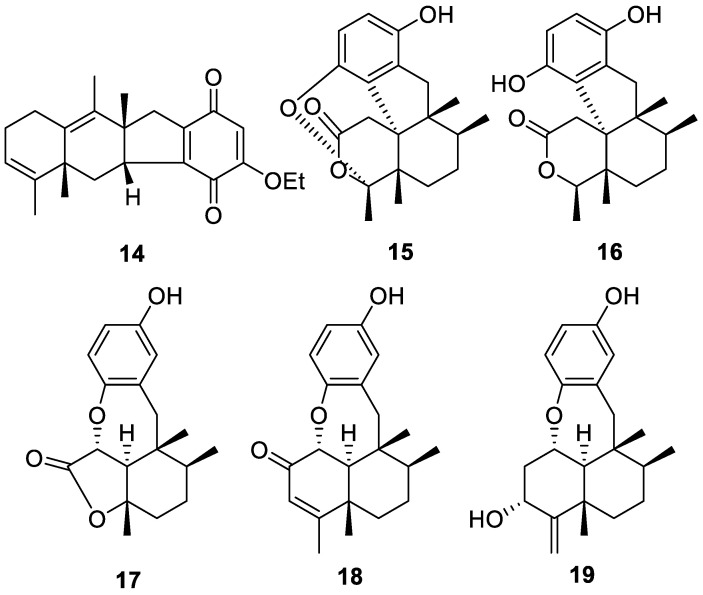
Structures of arenarialins A–F (**14**–**19**).

Arenarialins A–F (**14**–**19**, Figure 4) are six novel sesquiterpene quinone meroterpenoids isolated from *Dysidea arenaria*, a marine sponge collected from the South China Sea. The tetracyclic 6/6/5/6 carbon skeleton of arenarialin A (**14**) is unprecedented, while arenarialins B–D (**15**–**17**) feature rare secomeroterpene scaffolds. Upon evaluation of the compounds as anti-inflammatory agents, compounds **14**, **15**, **17**, and **18** displayed significant inhibitory activity against the production of TNF-α in LPS-induced RAW264.7 macrophages involved in the inflammatory cascade at various concentrations. Arenarialins B (**15**) and D–F (**17**–**19**), on the other hand, displayed a potent dose-dependent inhibition of the production of IL-6, a protein that regulates the immune system [11].

**Figure 5:**
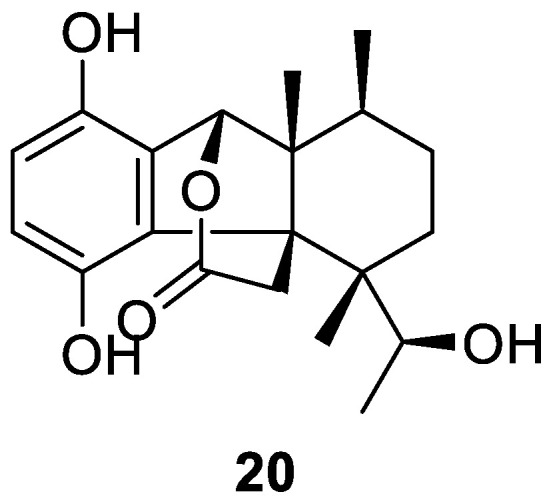
Structure of dysambiol (**20**).

Dysambiol (**20**, Figure 5) is a novel sesquiterpene hydroquinone isolated from a *Dysidea* sp. that was also collected in the South China Sea. This unusual compound was the first of its class to possess a 3,4-secomeroterpenoid scaffold featuring a rare lactone bridge. Compound **20** displayed potent suppression of the production of TNF-α, IL-1β, and IL-6 cytokines in activated RAW264.7 macrophage cells by regulating the NF-κB/MPAK signaling pathway for inflammatory responses. These results suggest that this unprecedented compound may be a good anti-inflammatory candidate for future analysis [12].

### 2.3. Diterpenoids

Diterpenoids are compounds with a 20-carbon skeleton consisting of four isoprene units. They can be either linear, bicyclic, tricyclic, tetracyclic, pentacyclic, or macrocyclic.

**Figure 6:**
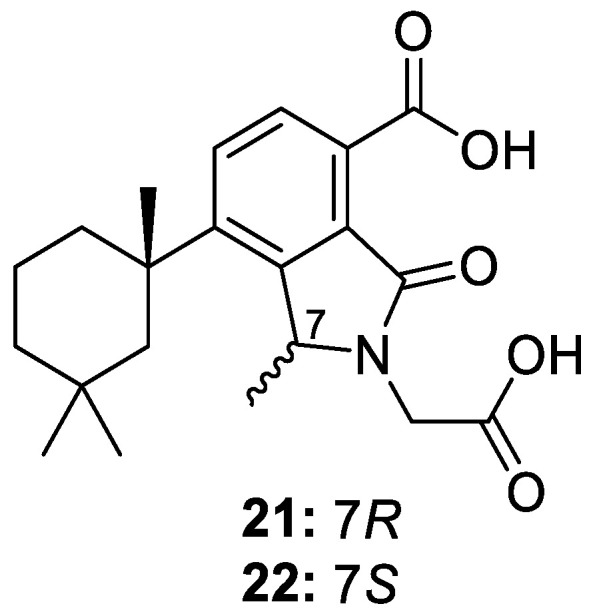
Structures of dendrillic acid A (**21**) and B (**22**).

Two novel isoindolinone nor-diterpenoids, dendrillic acids A (**21**) and B (**22**) (Figure 6), were isolated from a MeOH-dichloromethane (DCM) (1:1) extract of a sponge from the *Dendrilla* genus collected off the coast of Western Australia. Compounds **21** and **22** feature a unique glycine insertion at position C-7 but were deemed relatively inactive against various cancer cell lines. Dendrillic acid B (**22**) did display mild anti-protozoal activity against the parasite *Giardia duodenalis*, with an MIC of 50 μg/mL [13].

**Figure 7:**
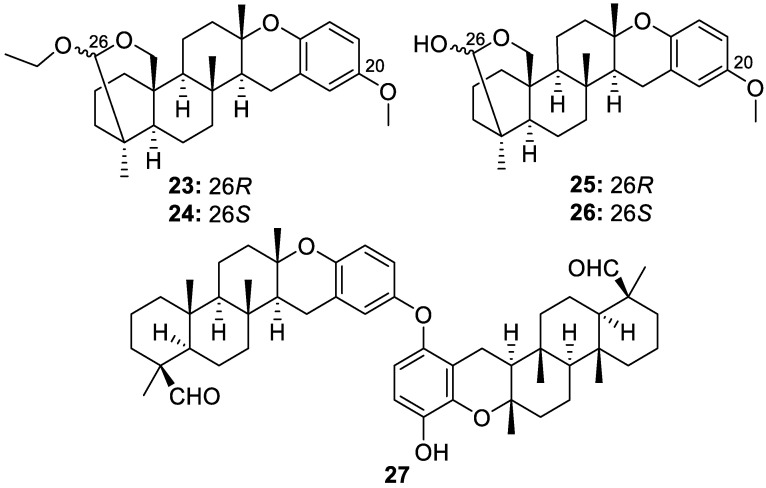
Structures of 20-*O*-methyl-26-*O*-ethylstrongylophorine-15 (**23**), 20-*O*-methyl-26-*O*-ethylstrongylophorine-16 (**24**), 20-*O*-methylstrongylophorine-15 (**25**), *O*-methylstrongylophorine-16 (**26**), and distrongylophorine A (**27**).

Bioassay-guided fractionation of a sponge from the *Petrosia* genus collected from the Solomon Islands yielded five novel strongylophorine meroditerpene analogs, 20-*O*-methyl-26-*O*-ethylstrongylophorine-15 (**23**, Figure 7), 20-*O*-methyl-26-*O*-ethylstrongylophorine-16 (**24**, Figure 7), 20-*O*-methylstrongylophorine-15 (**25**, Figure 7), *O-*methylstrongylophorine-16 (**26**, Figure 7), and distrongylophorine A (**27**, Figure 7). As stated in their report, compounds **23** and **24** may have been artifacts generated during isolation by ethoxylation at C-26 of **25** and **26** from the use of a solvent, such as ethyl acetate, during the isolation and purification process. The novel compounds did not display anti-tubercular activity in the initially performed assays [14].

**Figure 8:**
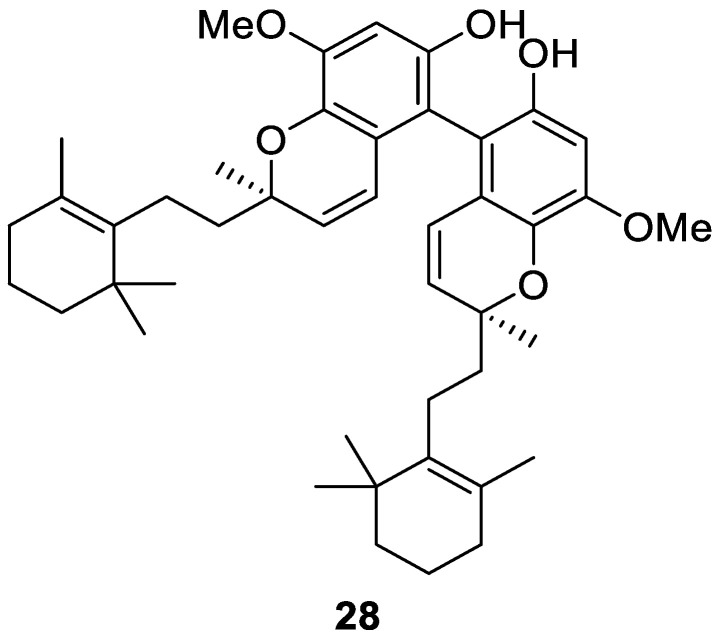
Structure of thorectidiol A (**28**).

Bioassay-guided fractionation of the MeOH extract of *Dactylospongia elegans*, collected off the coast of Papua New Guinea, led to the isolation of the racemic meroterpenoid thorectidiol A (**28**, Figure 8) as a dimeric diterpene. Compound **28** was active in an RBD-ACE2 assay for the selective inhibition of the SARS-CoV-2 viral spike protein, with an IC_50_ of 1.0 μM [15].

**Figure 9:**
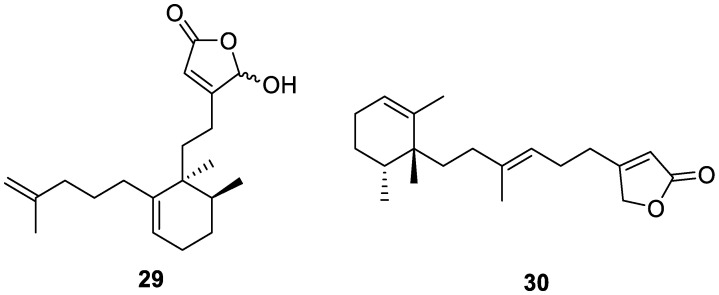
Structures of echinohalimane B (**29**) and oculatolide B (**30**).

Two new diterpenoids, echinohalimane B and subersin-type oculatolide B (**29** and **30**, respectively, Figure 9), were isolated from a MeOH extract of the freeze-dried sponge *Sarcotragus* sp. collected in the South China Sea. Echinohalimane B (**29**) demonstrated an unprecedented A-ring opening of halimane-type diterpenoid. Neither compounds **29** nor **30** displayed cytotoxic activities when tested [16].

**Figure 10:**
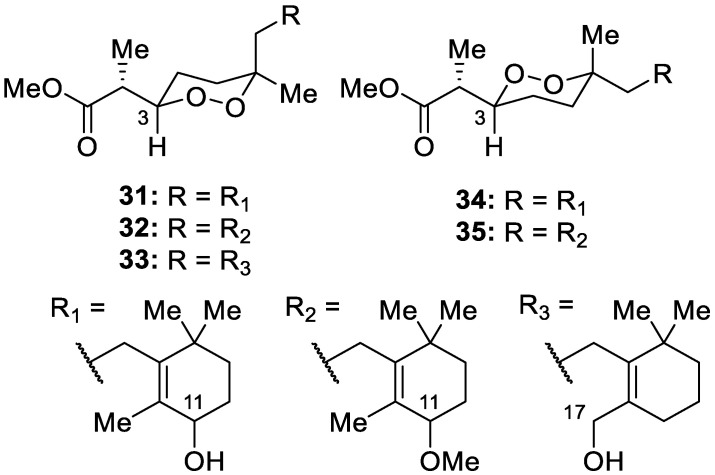
Structures of 11-hydroxy-diacarperoxide A (**31**) and its 3-epimer (**34**), 11-methoxy-diacarperoxide A (**32**) and its 3-epimer (**35**), and 17-hydroxy-nuapapuin A (**33**).

Bioassay-guided fractionation of a *Diacarnus spinipoculum* sponge extract collected in the Philippines yielded the isolation of five unprecedented nor-diterpene cyclic peroxides, 11-hydroxy-diacarperoxide A (**31**) and its 3-epimer (**34**), 11-methoxy-diacarperoxide A (**32**) and its 3-epimer (**35**), and 17-hydroxy-nuapapuin A (**33**) (Figure 10). The authors report that the epimeric mixture of 11-methoxy-diacarperoxide A is likely an artifact of the isolation process. Of these compounds, none displayed relevant inhibitory activity against hTRPA1 in pain and inflammatory pathways, despite the sponge extract showing an IC_50_ of 8.9 μg/mL in HEK-293 cells overexpressing hTRPA1 [17].

### 2.4. Sesterterpenoids

Terpenoids with five isoprene units (25 carbons), also named sesterterpenoids, are widely distributed in marine organisms, especially sponges [7].

**Figure 11:**
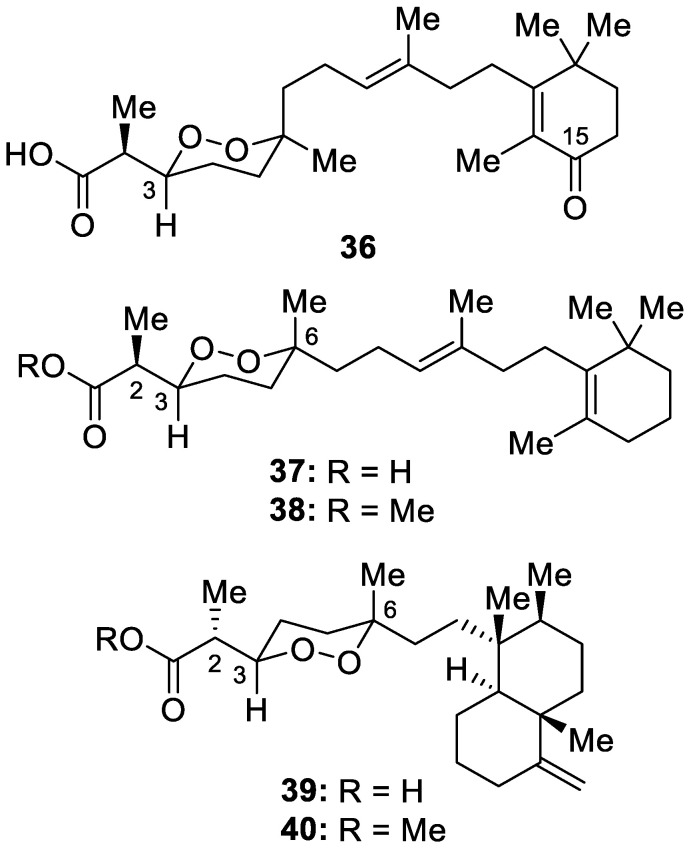
Structures of 15-carbony-(−)-3-*epi*-muqubilin (**36**), 2*S*,3*S*,6*R*-muqubilin (**37**) and its methyl ester (**38**), 2*R*,3*R*,6*S*-sigmosceptrellin (**39**) and its methyl ester (**40**).

Chemical investigation of an extract of a sponge identified as *Diacarnus spinipoculum* collected in the Philippines led to the isolation of five novel norsesterterpene cyclic peroxides, 15-carbony-(−)-3-*epi*-muqubilin (**36**), 2*S*,3*S*,6*R*-muqubilin (**37**) and its methyl ester (**38**), and 2*R*,3*R*,6*S*-sigmosceptrellin (**39**) and its methyl ester (**40**) (Figure 11). Following inhibitory testing against hTRPA1, 2*R*,3*R*,6*S*-sigmosceptrellin (**39**) was found to be the most potent, with an IC_50_ of 2.0 μM, while the other new compounds (**36**–**38**, **40**) also displayed some TRPA agonistic activities at 15.3, 5.6, 20.2, and 13.5 μM, respectively [17].

**Figure 12:**
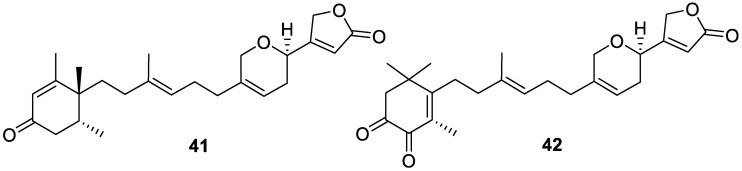
Structures of oshimalides A (**41**) and B (**42**).

Two sesterterpenes, oshimalides A and B (**41** and **42**, respectively, Figure 12), of the manoalide class, were isolated from a marine sponge of the *Luffariella* genus collected off the coast of southern Japan. Oshimalide A (**41**) displayed moderate antimicrobial activity against *Staphylococcus aureus*, with an MIC value of 51 μg/mL, while oshimalide B (**42**) was not tested due to its paucity [18].

**Figure 13:**
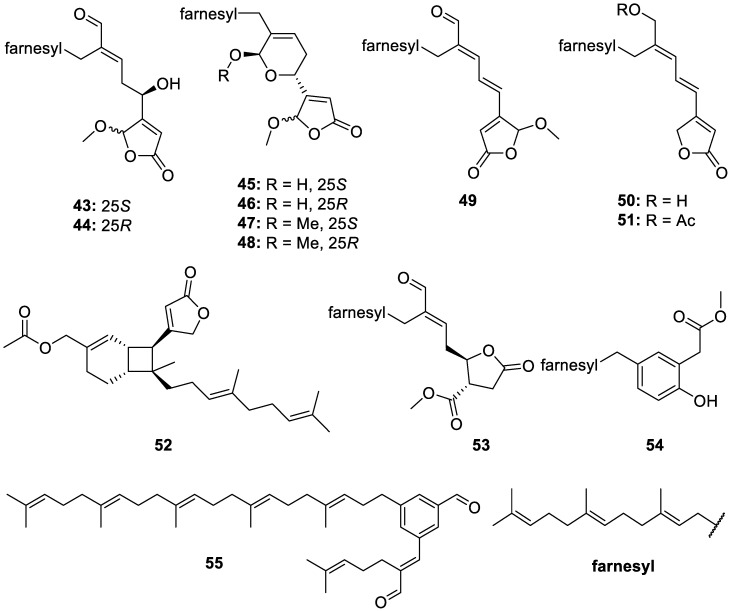
Structures of manoalide-type sesterterpenoid derivatives (**43**–**53**), a polyprenylphenol (**54**), a polyprenylbenzaldehyde (**55**), and farnesyl.

A frozen sample of *Luffariella variabilis* collected in the South China Sea was extracted and chemically investigated to yield thirteen novel manoalide-type sesterterpenoids, including eleven rare acyclic derivatives (**43**–**53**, Figure 13), one polyprenylphenol (**54**, Figure 13), and one polyprenylbenzaldehyde (**55**, Figure 13). Compounds **45**–**48** only have four other representative compounds from nature, representing a very rare class of sesterterpenoids, and compound **49** is the third known manoalide-type enantiomer from nature. Cytotoxicity against the K562 cell line was displayed for compounds **43**–**48**, the *S* and *R* enantiomers of **49**, and **55**, with IC_50_s of 4.0, 3.5, 2.9, 3.7, 3.2, 4.4, 4.5, 3.9, and 3.5 μM, respectively. Compounds **43**–**46** and **55** exhibited IC_50_ values of 6.1, 5.2, 4.8, 4.9, and 5.2 μM, respectively, against the H69AR cell line, while **48** had an IC_50_ of 4.3 μM against the MDA-MB-231 cell line [19].

**Figure 14:**
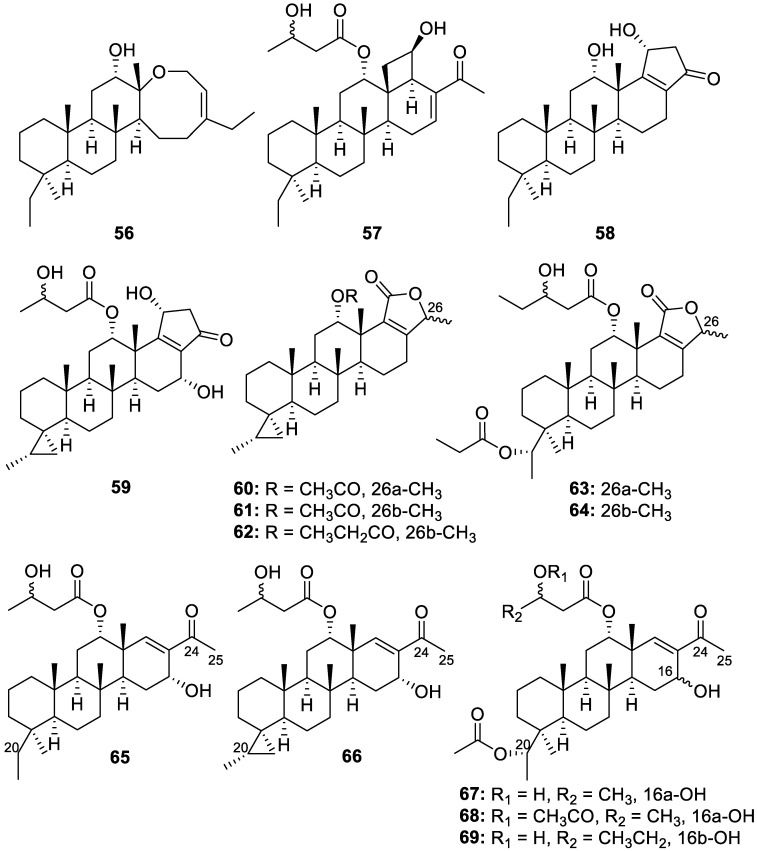
Structures of granulosane A (**56**), new 27-carbon sesterterpenes (**57**–**64**), and new 26-carbon 20,24-bishomo-25-norscalarane sesterterpenes (**65–69**).

Fourteen novel bishomoscalarane sesterterpenoids were isolated from *Dysidea granulosa* collected in the South China Sea. These include granulosane A (**56**) with a rare 6/6/6/8 tetracyclic skeleton, eight new 27-carbon sesterterpenes (**57**–**64**)**,** and five new 26-carbon 20,24-bishomo-25-norscalarane sesterterpenes (**65**–**69**) (Figure 14). Against the colon cancer (HCT116) and lung adenocarcinoma (A549) cell lines, compound **66** displayed anti-proliferative activity, with GI_50_ values of 6.4 and 8.1 μM, respectively [20].

**Figure 15:**
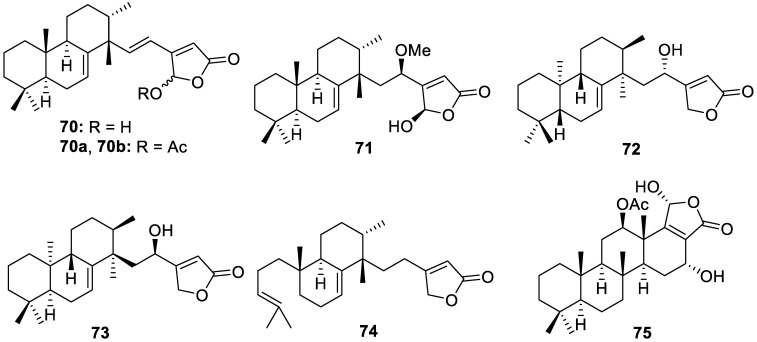
Structures of sarcotragusolides A−D (**70**–**73**), a γ-hydroxybutenolide sesterterpene derivative (**74**), and 12-β-*O*-acetylhyrtiolide (**75**).

Chemical and pharmacological investigations of an EtOAc extract from a *Sarcotragus* sponge species collected in the South China Sea led to the isolation of six new butanolide (**70**–**74**) and scalarane (**75**) sesterterpenes (Figure 15). Sarcotragusolides A–D (**70**–**73**) are rare cheilanthane sesterterpenes, while sarcotragusolides C (**72**) and D (**73**) display an unprecedented configuration inversion. Modest cytotoxic activity was exhibited by compounds **70a** and **70b** against K562 cells, with an IC_50_ of 4.38 and 2.91 μM, respectively. Sarcotragusolide B (**71**) displayed an IC_50_ value of 4.71 μM against the human pancreatic cancer (AsPC-1) cell line [16].

**Figure 16:**
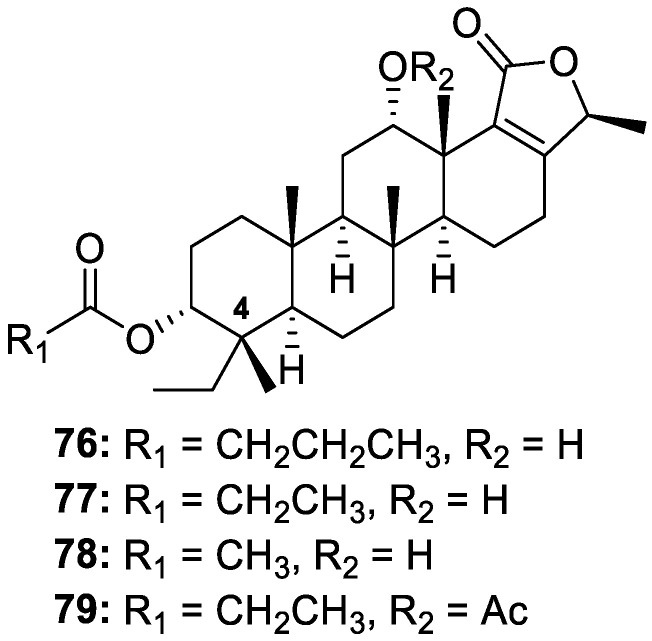
Structures of phyllolactones A−D (**76**–**79**).

Bioassay-guided fractionation and isolation performed on the DCM/MeOH extract prepared from the Australian sponge *Phyllospongia bergquistae* led to the isolation of bishomoscalarane phyllolactones A–D (**76**–**79**, Figure 16). The chemical structures of phyllolactones B (**77**) and C (**78**) were previously reported with the opposite stereochemical configuration at C-4 but were revised, as depicted below, based on a single-crystal X-ray diffraction analysis. Compounds **76**–**79** displayed mild anthelmintic activities by inhibiting the motility of exsheathed third-stage larvae (xL3s) of *H. contortus* by ≥70% within 90 h at concentrations between 5.3 and 10.1 μM [21].

**Figure 17:**
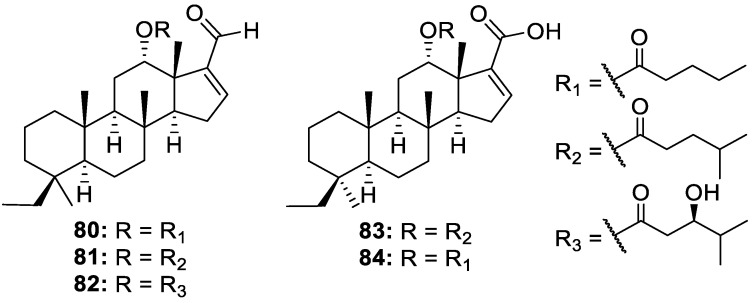
Structures of phyllospongianes A−E (**80**–**84**).

Five related compounds bearing a new 6/6/6/5 tetracyclic dinorscalarane scaffold, phyllospongianes A–E (**80**–**84**, Figure 17), were isolated from the *Phyllospongia foliascens* sponge collected from the South China Sea. Phyllospongianes A (**80**), B (**81**), and D (**83**) exhibited antibacterial activities against *V. vulnificus*, *V. parahemolyticus*, *E. coli*, *E. faecalis*, *B. subtilis*, and *P. aeruginosa*, with MICs ranging from 1 to 8 μg/mL. The novel compounds displayed mild cytotoxic activity, but phyllospongiane C (**82**) exhibited significant cytotoxicity on enzalutamide-resistant prostate cancer (C4-2-ENZ), breast adenocarcinoma (MCF-7), non-small cell lung cancer (NCI-H460), and colorectal adenocarcinoma (HT-29) cell lines, with IC_50_ values of 0.7, 1.1, 2.0, and 1.2 μM, respectively [22].

**Figure 18:**
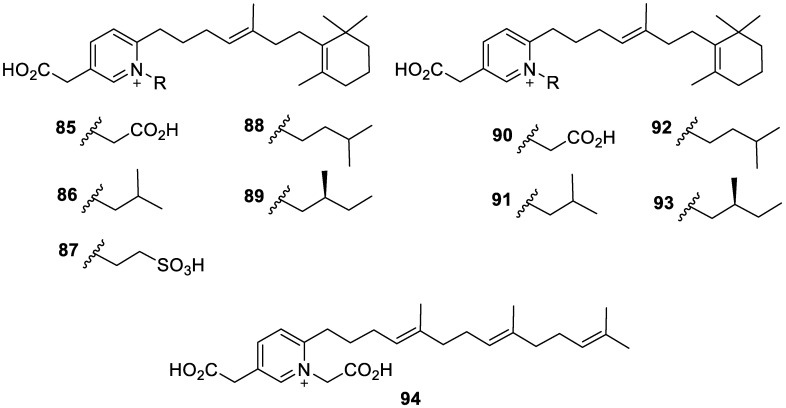
Structures of coscinoderines A–J (**85–94**).

Coscinoderines A–J (**85**–**94**, Figure 18) are ten new norsesterterpene alkaloids isolated from the MeOH/DCM (2:1) extract of a marine sponge *Coscinoderma bakusi* collected off an island in the Federated States of Micronesia. Although the crude extract did display TRPA1 inhibition in HEK-293 cells at a concentration of 10 μg/mL, the isolated novel compounds did not display relevant cytotoxic or antibacterial activity. The isolated coscinoderines with an unprecedented 1,2,5-trisubstituted pyridinium scaffold and terpene unit at C-2 added to the lists of secondary metabolites that contain pyridinium and alkaloids from *C. bakusi* [23].

### 2.5. Triterpenoids and Steroids

#### 2.5.1. Triterpenoids

Triterpenoids (30 carbon skeleton) comprise six isoprene units derived from acyclic hydrocarbon and squalene and have relatively complex cyclization patterns. Most triterpenes contain either alcohols, carboxylic acids, or aldehydes as functional modifications [7].

**Figure 19:**
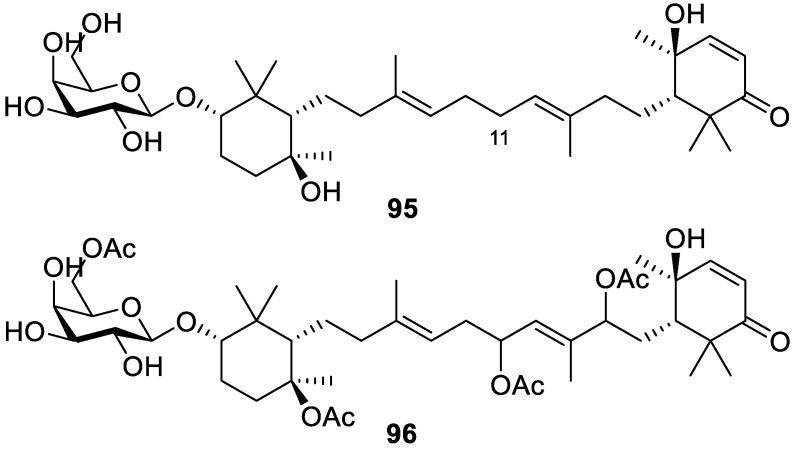
Structures of melophluosides A (**95**) and B (**96**).

Melophluosides A and B (**95** and **96**, respectively, Figure 19) are two new triterpene galactosides that were isolated from the marine sponge *Melophlus sarasinorum*, collected in Indonesia. Compound **95** was the first reported compound in the pouoside class, triterpenoid saponins found in marine sponges, that lacks an oxygenated group on C-11. Although the discovered compounds did not display antimicrobial properties against bacteria or yeast, compounds **95** and **96** showed moderate cytotoxicity against immortal human cells (HeLa), with IC_50_ values of 11.6 and 9.7 μM, respectively [24].

**Figure 20:**
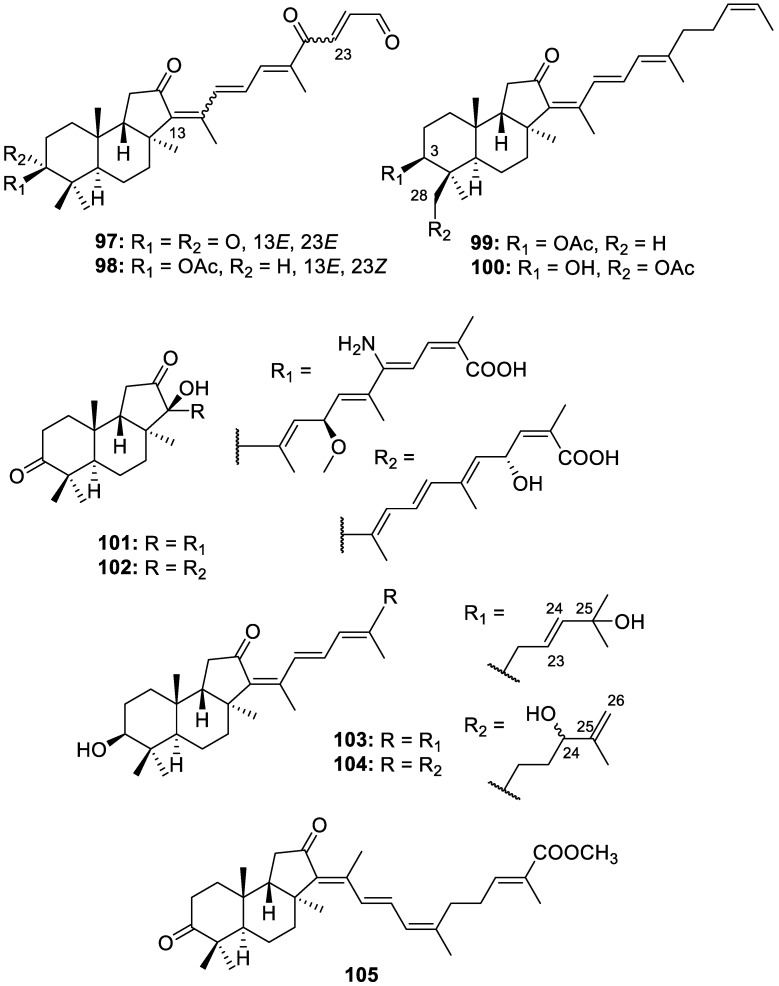
Structures of 13-(*E*)-geoditin A (**97**), 13-(*E*)-isogeoditin B (**98**), 3-acetylstelliferin D (**99**), 28-acetylstelliferin D (**100**), hainanstelletin A (**101**) and B (**102**), 23,24-ene-25-hydroxystelliferin D (**103**), 25,26-ene-24-hydroxystelliferin D (**104**), and hainanstelletin C (**105**).

Nine novel isomalabaricane triterpenoids, 13-(*E*)-geoditin A (**97**), 13-(*E*)-isogeoditin B (**98**), 3-acetylstelliferin D (**99**), 28-acetylstelliferin D (**100**), hainanstelletins A and B (**101** and **102**, respectively), 23,24-ene-25-hydroxystelliferin D (**103**), 25,26-ene-24-hydroxystelliferin D (**104**), and hainanstelletin C (**105**) (Figure 20), were isolated from the *Rhabdastrella globostellata* sponge collected in the South China Sea. Hainanstelletin A (**101**) was the first nitrogenous isomalabaricane reported to date. 13-(*E*)-geoditin A (**97**) and 13-(*E*)-isogeoditin B (**98**) showed significant antibacterial activity against *S. pyogenes*, with MICs of 1.8 and 1.0 μg/mL, respectively, and moderate antibacterial activity against *S. aureus* [25].

**Figure 21:**
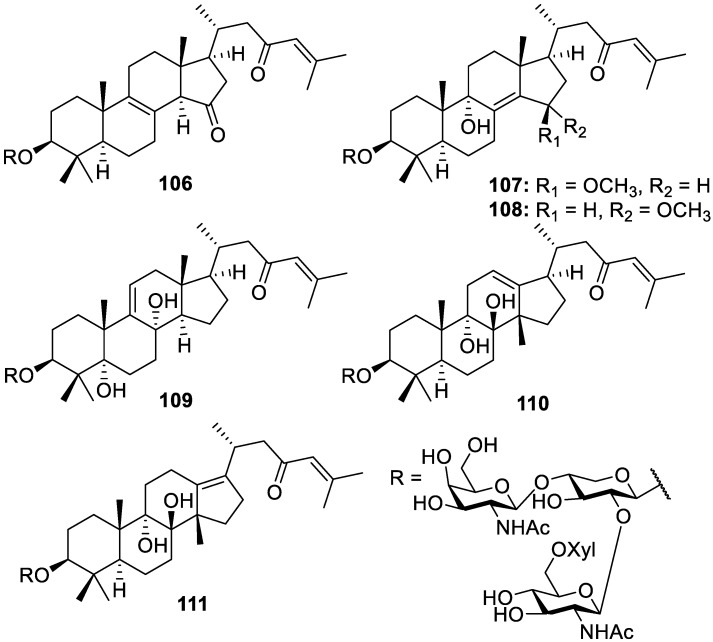
Structures of sarasinosides C_4–9_ (**106**–**111**).

Six novel 30-norlanostane saponin triterpenoid derivatives, sarasinosides C_4–9_ (**106**–**111**, Figure 21), were isolated from the sponge identified as *Melophlus sarasinorum* and collected off the coast of Papua New Guinea. The novel metabolites show various oxidation patterns of the aglycone in addition to characteristic side chains and carbohydrate moieties of the sarasinoside C series (Figure 21). No significant cytotoxicity was observed by the sarasinosides in the tested MTT assays against A549, metastatic melanoma (A2058), hepatocyte carcinoma (HepG2), MCF-7, and pancreatic carcinoma (MiaPaCa) cell lines [26].

#### 2.5.2. Steroids

Steroids are a type of degraded triterpene categorized by their cyclopentane perhydrophenanthrene ring systems [7].

**Figure 22:**
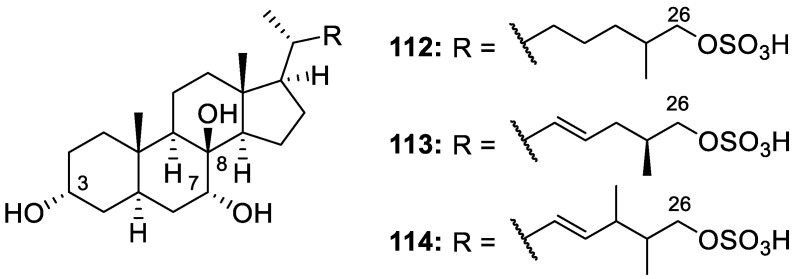
Structures of lamellosterols A–C (**112**–**114**).

Three new sulfated polyoxygenated sterols, lamellosterols A−C (**112**–**114**, Figure 22), were isolated from the MeOH extract of freeze-dried *Lamellodysidea* cf. *chlorea* using bioassay-guided fractionation. The 3α,7α,8β-oxidation pattern of the isolated lamellosterols had never been reported in sponges, and the 8β-hydroxylation was very rare in sponge sterols. Compounds **112**–**114** displayed potent anti-prion activity against the [PSI+] yeast prion, with EC_50_ values of 12.7, 13.8, and 9.8 μM, respectively. Lamellosterol A (**112**) also displayed potential for neuroprotective activity against Parkinson’s disease in a thioflavin T (ThT) assay by binding to α-synuclein in vitro and inhibiting its aggregation (~70% reduction in ThT fluorescence) [27].

**Figure 23:**
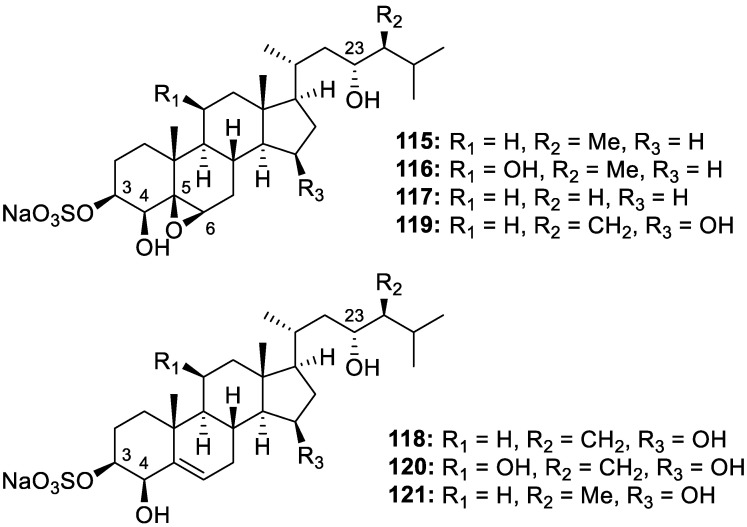
Structures of gracilosulfates A–G (**115**–**121**).

Gracilosulfates A–G (**115**–**121**, Figure 23) were seven novel polyoxygenated steroids isolated from the sponge *Haliclona gracilis* collected off the Russian coast in the Northwestern Pacific Ocean. Although sulfate-containing molecules are abundant from marine sources, sulfated sterols are rare from sponges of the *Haliclona* genus. This new group of monosulfated steroids contains the common structural motifs of 3β-*O*-sulfonato, 5β,6β-epoxy, or 4β,23-dihydroxy substitution patterns. Gracilosulfate G (**121**) exhibited weak cytotoxicity against hormone-independent prostate cancer cells, with an IC_50_ = 64.4 μM, while the other isolated compounds were found effective in the concentration-dependent inhibition of a prostate-specific antigen (PSA) in human prostate cancer (22Rv1) cells, indicating that these compounds may inhibit androgen receptor (AR) signaling [28].

## 3. Alkaloids

### 3.1. Brominated Alkaloids

**Figure 24:**
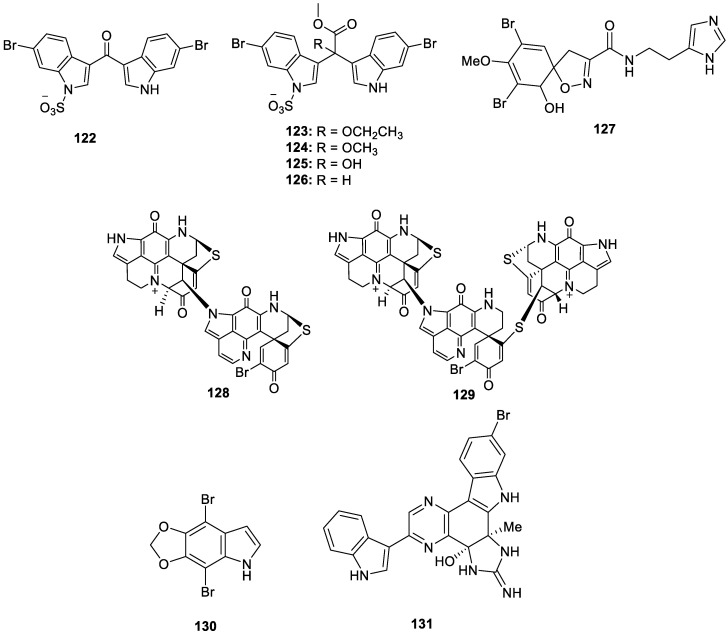

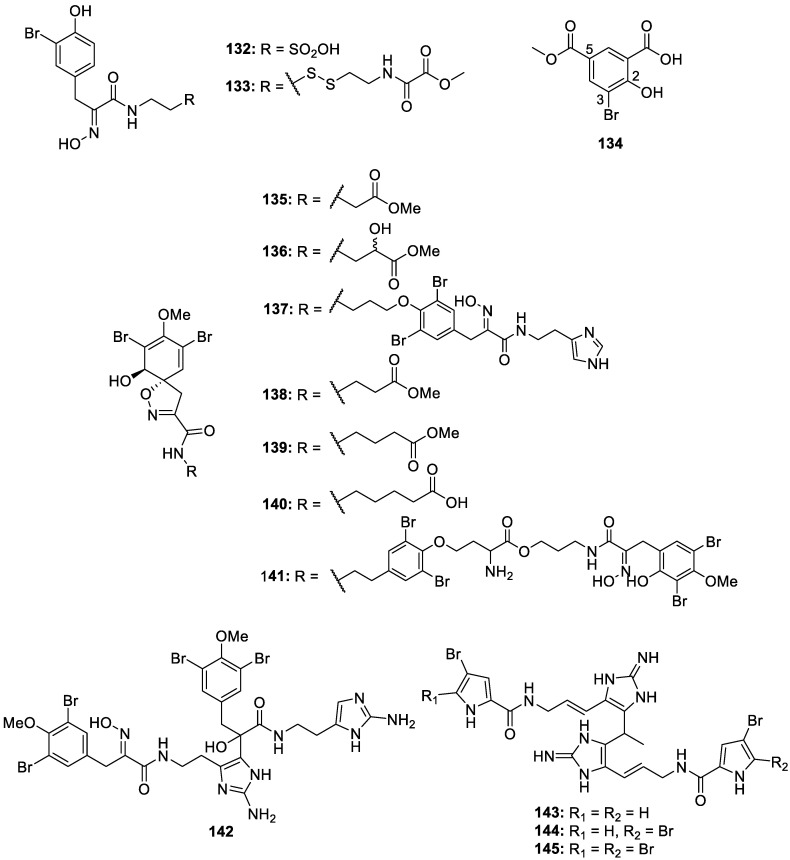

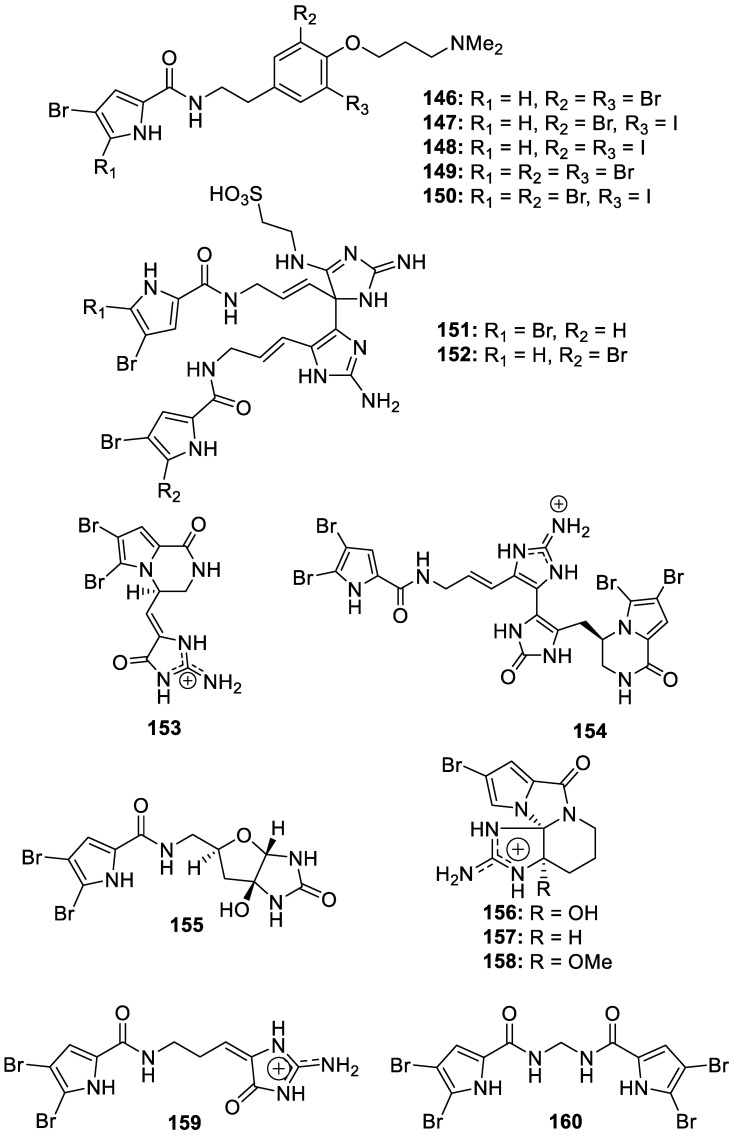

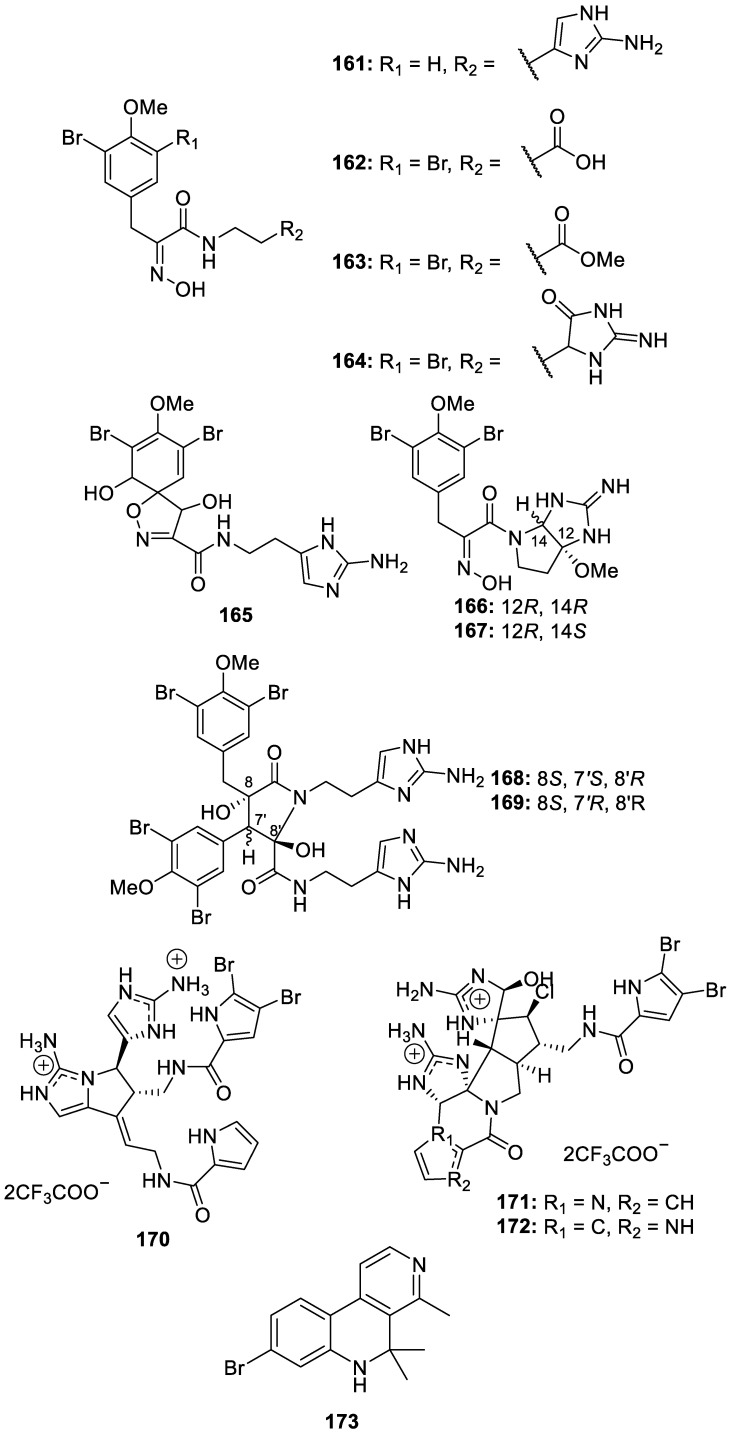
Structures of brominated alkaloids (**122**–**173**).

According to Dr. S. William Pelletier, alkaloids are “cyclic compound[s] containing nitrogen in a negative oxidation state, which is of limited distribution among living organisms” [29]. In addition to new isolation efforts, some of the recent literature surrounding alkaloids has covered topics such as structural revisions or the in vivo testing of previously described sponge-derived natural products. For example, the structures of echinosulfone A (**122**, Figure 24), a dibrominated bis-indole alkaloid, and echinosulfonic acids A–D (**123**–**126**, Figure 24) from the Australian sponge in the *Crella* genus, were revised in a recent article after reanalysis of the spectrometric data [30]. Many brominated alkaloids have been isolated from various species of marine and coastal sponges and investigated for their pharmacological properties. Most of these compounds display appealing biological activities such as cytotoxicity, antibacterial properties, enzyme inhibition, or neuroprotective abilities. Aerophobin-1 (**127**), a known bromotyrosine derivative from *Aplysina aerophoba*, was recently highlighted as a promising pro-osteogenic (anti-osteoporotic) candidate for regenerative medicine, representing the first report of effects on bone development from a marine alkaloid of its class [31]. In terms of recently isolated metabolites, didiscorhabdin (**128**) and tridiscorhabdin (**129**) are new discorhabdin-type alkaloids from the sponge *Latrunculia biformis* collected from the Weddell Sea of Antarctica. These novel compounds are the first examples of a direct C−N bridge in discorhabdin oligomers, and tridiscorhabdin (**129**) displayed potent cytotoxic activity against the HCT-116 cancer cell line, with an IC_50_ value of 0.31 μM [32]. Isolated from a marine sponge of the *Psammocinia* genus, amakusamine (**130**) is the first methylenedioxy dibromoindole to exhibit anti-osteoporosis activity, with an IC_50_ value of 10.5 μM against RAW264 macrophage cells [33]. Bioassay-guided isolation of *Myrmekioderma* sp. yielded a brominated bis-indole with a new carbon skeleton, myrindole A (**131**), that displayed antimicrobial properties against both *E. coli* (Gram-negative) and *B. subtilis* (Gram-positive), with MIC values of 37.5 and 18.5 μM, respectively [34]. Several bromotyrosine alkaloids were also isolated from *Aplysinella rhax*, including three analogs of psammaplin that have never before been found in nature, psammaplin O (**132**), psammaplin P (**133**), and 3-bromo-2-hydroxy-5-(methoxycarbonyl)benzoic acid (**134**). This group also provides the first report of antimalarial activity against *T. cruzi* and *P. falciparum* from this scaffold [35]. Although the MeOH extract of a *Aplysina lacunose* sponge showed α-chymotrypsin enzyme inhibition, the isolated bromotyrosine spiroisoxazoline alkaloids, named lacunosin A (**135**), lacunosin B (**136**), and desaminopurealin (**137**) were not deemed active as protease inhibitors [36]. Four additional spiroisoxazoline alkaloids, purpuroceratates A and B (**138** and **139**), purpuroceratic acid C (**140**), and ningalamide A (**141**) alongside the dimerized amide, ningalamide B (**142**), were isolated from a *Pseudoceratina* cf. *verrucosa* sponge near Western Australia. Despite the novel isolates not displaying any relevant activity, the researchers report potential for these compounds to serve as a new scaffold for acetylcholinesterase inhibitors in association with Alzheimer’s disease [37]. Novel alkaloidal metabolites were isolated from *dispar* and *oroides* species of the *Agelas* genus but were not tested for pharmacological activity. From *A. dispar*, ten bromopyrrole derivatives were identified as disparamides A–C (**143**–**145**), dispyrins B–F (**146**–**150**), and nagelamides H2 (**151**) and H3 (**152**) [38]. Eight bromopyrrole derivates were isolated from *A. oroides* and named agesamine C (**153**), dioroidamide A (**154**), slagenin D (**155**), (−)-monobromoagelaspongin (**156**), (−)-11-deoxymonobromoagelaspongin (**157**), (−)-11-*O*-methylmonobromo-agelaspongin (**158**, Figure 24), E-dispacamide (**159**, Figure 24), and pyrrolosine (**160**) [39]. Mild antifungal activity was displayed by novel bromotyrosine alkaloids, debromoianthelline (**161**), pseudoceratinic acid (**162**), methylpseudoceratinate (**163**), 13-oxo-ianthelline (**164**), 7-hydroxypurealidin J (**165**), and aiolochroiamides A–D (**166**–**169**) and isolated from the Bahamian *Aiolochroia crassa* sponge. Of these nine isolates, aiolochroiamides C (**168**) and D (**169**) moderately inhibited *Candida* and *Cryptococcus* spp. but were not deemed responsible for the antimicrobial activity of the *A. crassa* MeOH extract [40]. Futunamine (**170**), an unprecedented alkaloid featuring a pyrrolo [1,2-*c*]imidazole core, and two other dimeric pyrrole 2-aminoimidazole derivatives identified as debromokonbu’acidin (**171**) and didebromocarteramine (**172**) were isolated from *Stylissa aff. carteri* collected near the Futuna Islands. The compounds were tested for their anti-inflammatory and neuroprotective properties on the human neuroblastoma (SH-SY5Y) and microglia (BV2) cellular models. Futunamine (**170**) and debromokonbu’acidin (**171**) were found to reduce ROS production by 35% at all concentrations (cell death decreased by compound **170** at 10 μM) after the treatment of cells with oxidant TBHP [41]. Veranamine (**173**), a previously reported alkaloid from the Florida sponge *Verongula rigida* with a unique benzo[*c*][2,7]naphthyridine scaffold, was pharmacologically evaluated for its potential anti-depressant properties based on its structural similarities to compounds with known neurological activity. Upon isolation, veranamine (**173**) demonstrated a relatively high binding affinity for 5HT2B and sigma-1 receptors, with *K_i_* values of 390 and 560 nM, respectively. This natural product may serve as a lead scaffold for the future development of psychiatric medications with unique receptor-binding profiles [42].

### 3.2. Non-Brominated Alkaloids

**Figure 25:**
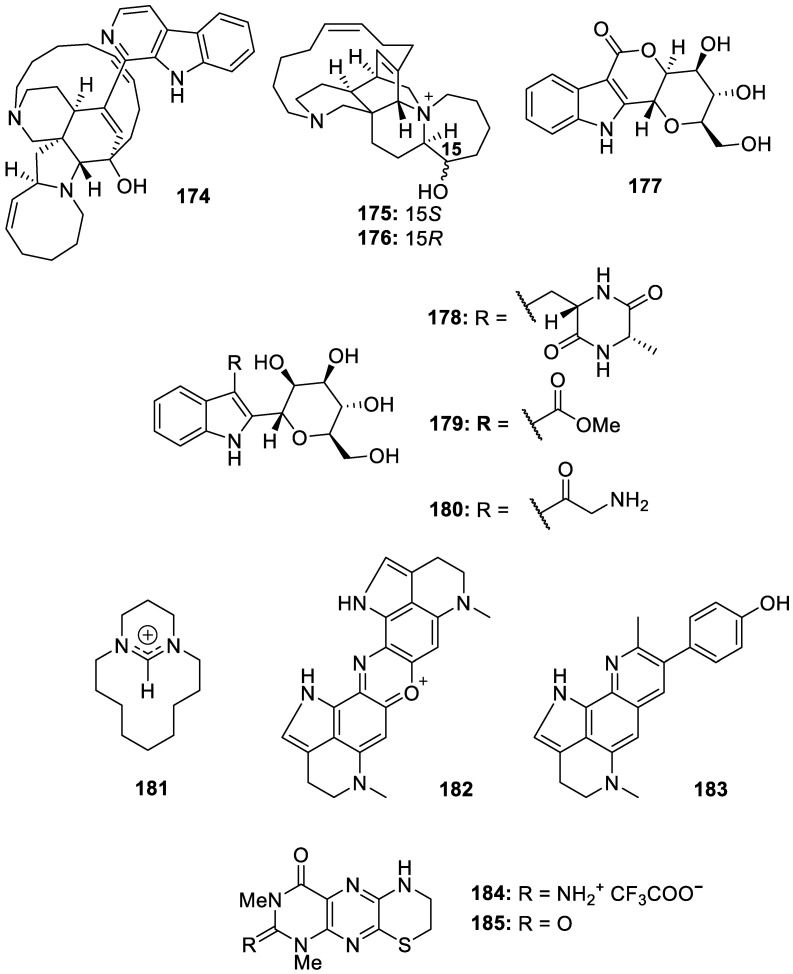
Structures of non-brominated alkaloids (**174**–**185**).

There have also been several non-brominated alkaloids isolated from the Indo-Pacific and marine sponges that possess potential for scaffolds as new therapeutic agents in drug development. Manzamine A (**174**, Figure 25), isolated from *Haliclona*, displayed anti-proliferative activity against cervical cancer cell lines (C33A, HeLa, SiHa, and CaSki) at concentrations up to 4 μM by decreasing the levels of SIX1 and CK2α proteins [43]. Extraction efforts of an *Amphimedon* sp. marine sponge led to the isolation of two novel manzamine-related alkaloids, zamamiphidins B (**175**, Figure 25) and C (**176**, Figure 25). Zamamiphidins B (**175**) and C (**176**) represented a unique fused diazahexacylic ring system and had weak acetylcholinesterase inhibitory activity, with IC_50_ values of 0.35 and 0.47 mM, respectively [44]. In vivo hepatoprotective activity was exhibited by indole-*C*-mannopyranoside alkaloids, petrosins A–D (**177**–**180**, Figure 25), isolated together with haliclorensin D (**181**, Figure 25), a new diamine alkaloid, from *Neopetrosia chaliniformis*. The zebrafish model revealed that petrosins A (**177**), B (**178**), and D (**180**) demonstrated moderate hepatoprotective properties at 20 μM concentrations compared to the positive control [45]. Two additional analogs of pyrroloiminiquinones, zyzzamines A and B (**182** and **183**, respectively, Figure 25), were isolated upon reinvestigation of an Indo-Pacific sponge identified as *Zyzzya fuliginosa*. Although the zyzzamines failed to display significant bioactivity, the authors report potential for these pyrrole–quinoline alkaloids as a prospective scaffold for cytotoxic compounds against PANC-1 cells [46]. Furthermore, despite their lack of biological activity, two new fluorescent pteridine alkaloids named tedaniophorbasins A and B (**184** and **185**, Figure 25) were isolated from the Australian sponge *Tedaniophorbas ceratosis* and are suggested to play a role in bioluminescence as luminophores [47].

## 4. Peptides

**Figure 26:**
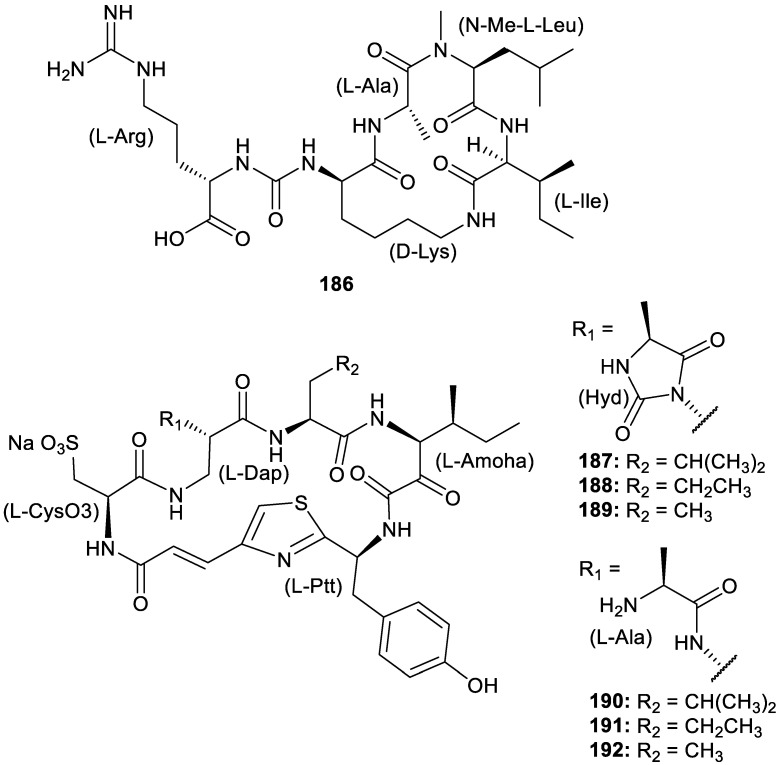

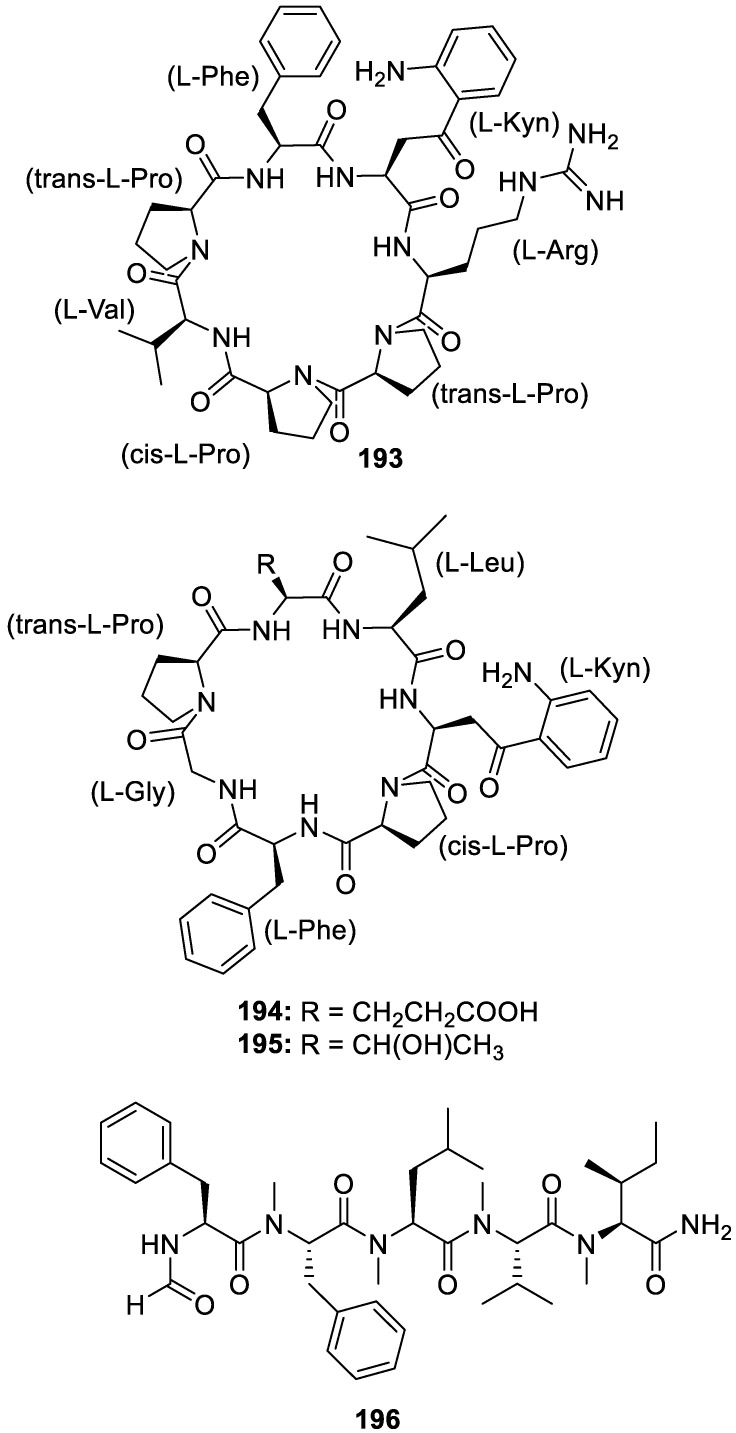

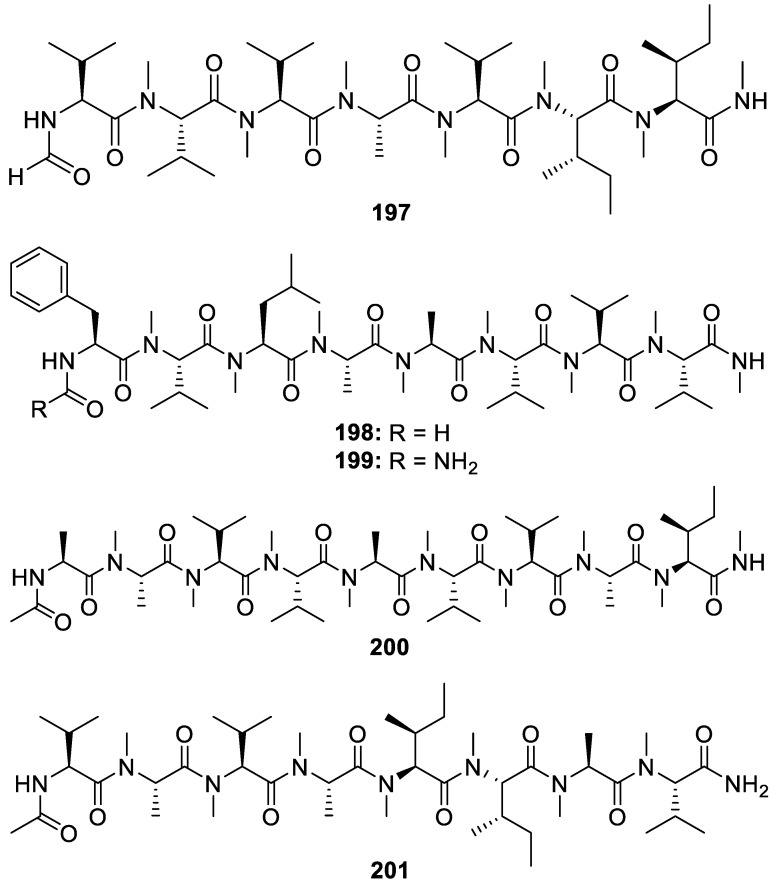

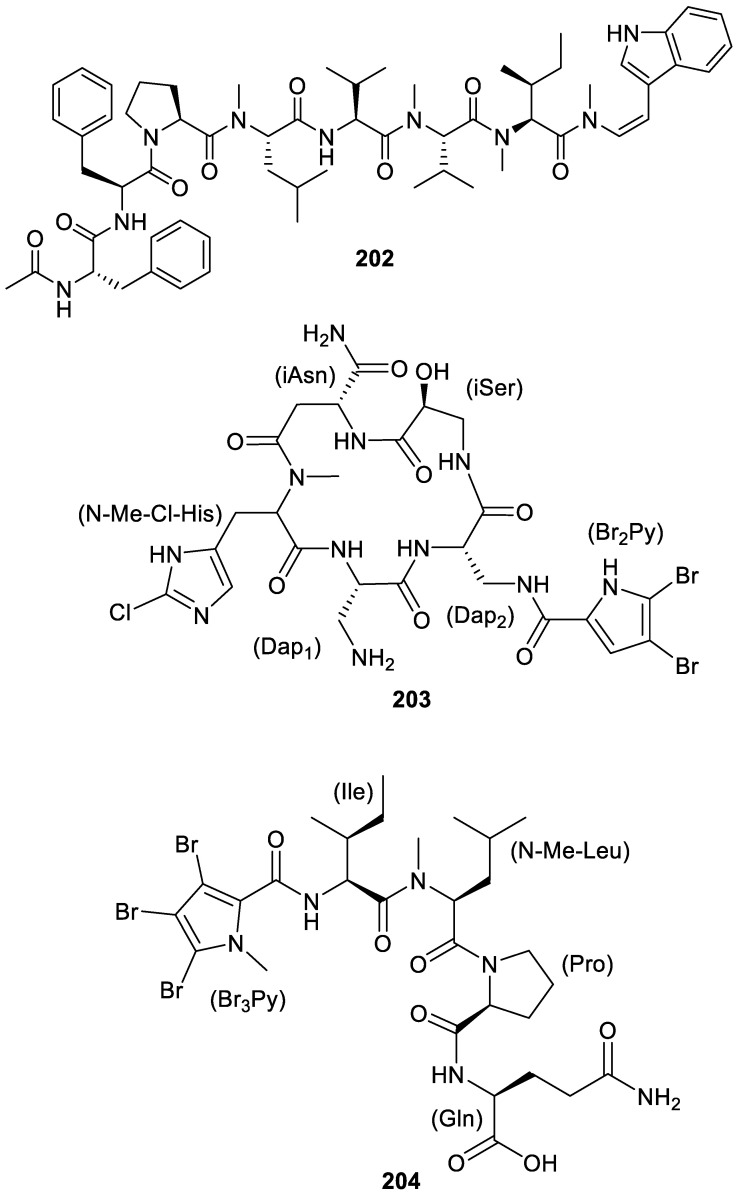
Structures of peptides (**186–204**).

Peptides isolated from natural sources display promising bioactivities for pharmacological drug development. A novel cyclic peptide, homophymamide A (**186**, Figure 26), has been isolated from a species of marine sponge from the *Homophymia* genus and found to inhibit carboxypeptidase B, with an IC_50_ value of 0.59 μM [48]. New cyclic peptides were also isolated from the Australian sponge *Theonella* sp. and identified as cyclotheonellazoles D–I (**187–192**, Figure 26). The cyclotheonellazoles displayed potent nanomolar inhibition of serine protease elastase, with IC_50_s ranging from 16.0 nM to 61.8 nM) and weak enzyme inhibitory activity against chymotrypsin, with IC_50_ values ranging from 0.73 to 2.7 μM [49]. Three new kynurenine-containing cycloheptapeptides elucidated as phakefustatins A–C (**193–195**, Figure 26) were discovered from *Phakellia fusca*. Phakefustatin A (**193**) was identified to inhibit cancer cell growth by modulating RXRα as part of the PI3K/Akt signaling pathway by exhibiting cytotoxicity against MCF-7, HeLa, and NCI-H460 cell lines, with IC_50_ values of 3.4, 6.2, and 7.1 μM, respectively [50]. Novel peptides isolated from the Antarctic sponge *Inflatella coelosphaeroides* were reported and identified as shagamides A–F (**196**–**201**, Figure 26) and friomaramide B (**202**). They exhibited high degrees of *N*-methylation. The shagamides containing an *N*-terminal phenylalanine residue (A (**196**), C (**198**), and D (**199**)) exhibited micromolar activity against three blood-stage *P. falciparum* strains (NF54, Dd2, and 3D7), demonstrating potential for future optimization as antimalarial metabolites [51]. Based on known sequencing data for barrettides A and B, a research group identified five new barrettide sequences, barrettides C–G, as part of this peptide family uniquely produced by the demosponge *Geodia barretti*. Anti-biofouling activity against larva of bay barnacle *Amphibalanus improvisus* was displayed by barrettide C (NVVPCFCVEDETSGAKTCIPDNCDASRGTNP, disulfide connectivity I–IV, II–III) with an IC_50_ of 0.64 μM [52]. Lastly, despite not displaying any relevant cytotoxicity, an unprecedented dibromopyrrole cyclopeptide with a chlorohistidine ring, haloirciniamide A (**203**), and the rare tribromopyrrole linear peptide, seribunamide A (**204**), were isolated from a sponge of the *Ircinia* genus, adding to the library of novel natural product skeletons [53].

## 5. Miscellaneous Constituents

**Figure 27:**
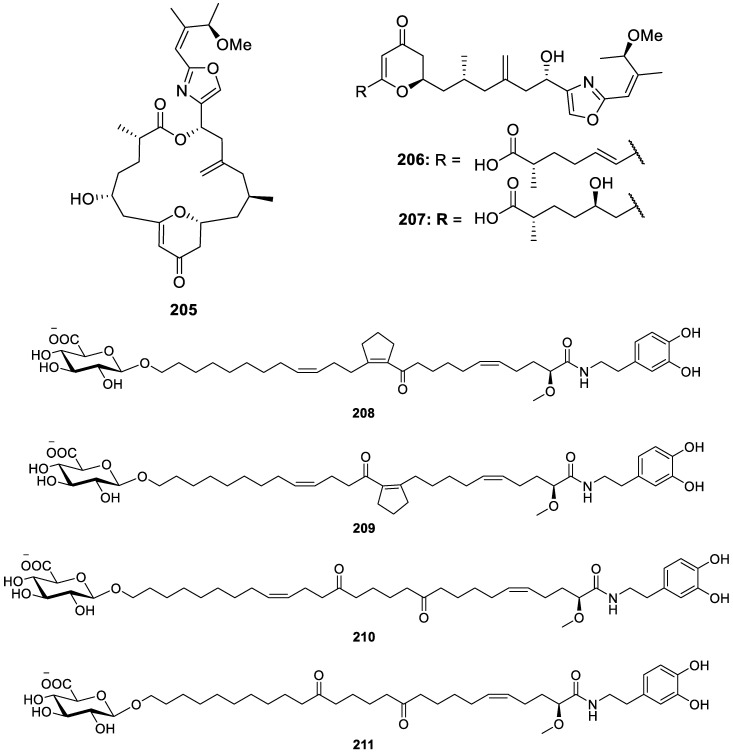

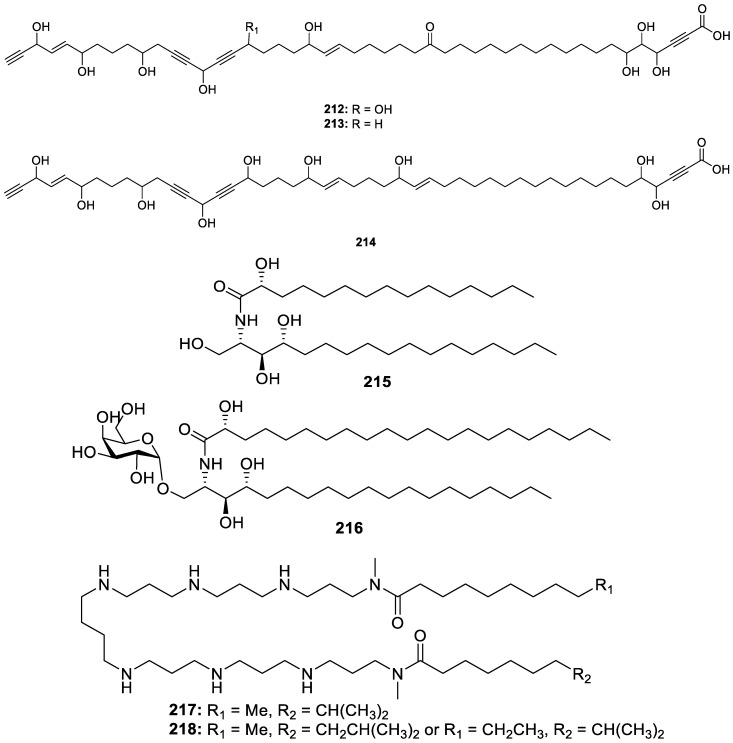
Structures of miscellaneous constituents (**205**–**218**).

Other chemical constituents from sponges have also been found to portray biological activity, like lipids, polyamines, polymers, and macrolides. Micromolar cytotoxic activity against A549, HT-29, MDA-MB-231, and pancreas (PSN-1) tumor cells was exhibited by enigmazoles C and D (**205** and **206**, respectively) of the new macrolide lactone analogs named enigmazoles C–E (**205**–**207**, Figure 27) from a species of the *Homophymia* genus [54]. New glycosylated fatty acid amides, toporosides A–D (**208**–**211**), were isolated from the sponge *Stelodoryx toporoki*, two of which, toporosides A (**208**) and B (**209**), feature a unique cyclopentenyl moiety in the polymethylene chain. Furthermore, toporosides A (**208**), C (**210**), and D (**211**) displayed cardioprotective activity by increasing the survival of TNF-α-treated H9c2 cardiomyocytes by 23%, 25%, and 18%, respectively [55]. Extraction of a *Haliclona* sp. sponge collected from Mayotte yielded three new long-chain highly oxygenated polyacetylenes named osirisynes G–I (**212**–**214**, Figure 27), of which osirisynes G (**212**) and I (**214**) mildly inhibited proteasome in a fluorescence intensity biological assay [56]. Stylissamide A (**215**, Figure 27), a ceramide, and stylissoside A (**216**, Figure 27), a cerebroside, are two new bioactive lipids discovered through bioassay-guided isolation of a crude methanol extract of *Stylissa carteri*, a Red Sea sponge. Both unprecedented sphingolipids exhibited potent cytotoxic activity against the MCF-7 and HepG2 human cancer cell lines, with stylissamide A (**215**) exhibiting stronger properties towards MCF-7 (IC_50_ = 30.5 μM) and stylissoside A (**216**) being more active against HepG2 cells (IC_50_ = 21.1 μM) [57]. Lastly, two novel amphiphilic polyamines, identified as aaptolobamines A (**217**, Figure 27) and B (**218**, Figure 27), were isolated using a bioassay-guided fractionation of an *Aaptos lobata* extract prior to being tested for a broad range of relevant pharmacological properties, including cytotoxicity, antimicrobial activity, and enzyme inhibition. Aaptolobamines A (**217**) and B (**218**) were active against cancerous prostate cells (PC-3), with IC_50_ values of 3.4 and 4.1 μM, respectively, displayed moderate antimicrobial activity against *S. aueus* strains, and inhibited α-synuclein amyloid aggregation in Parkinson’s disease, which suggests the use of polyamines for the treatment of neurodegenerative disorders [58].

## 6. Conclusions

This review covered 218 compounds with unprecedented structures and promising biological activity isolated from sponges over the past three years (2020–2023). They included 121 terpenoids, 52 alkaloids, 19 peptides, and 14 miscellaneous compounds. These results display the wide range of prospective pharmacological properties of compounds discovered from marine sources and encourage the need for the continued investigation of marine natural products. We present evidence of potential for advancements from sponges with these reported activities: cytotoxicity, antimicrobial/antibacterial, anti-proliferative, anti-protozoal, anti-tubercular, antimalarial, anthelmintic, anti-viral, anti-prion, anti-biofilm, anti-inflammatory, anti-depressant, neuroprotective, cardioprotective, and enzyme inhibitory. As cytotoxicity was the most evaluated and reported biological activity, we have summarized the most active compounds regarding cytotoxicity (IC_50_ ≤10 µM) isolated during the specified period (Table 1). The represented compounds demonstrate new lead scaffolds for development and optimization in their respective categories for natural product research and drug discovery. This review also highlights the unique structural features of the isolated constituents and draws attention to the complexity of the biological pathways of organisms in the marine environment.

## Figures and Tables

**Table 1:** List of cytotoxic compounds isolated with IC_50_ values of ≤10 µM.

Compound	Bioassay	Activity (µM)	Origin (Sponge)	Collection Site	Compound Type	Reference
pseudoceranoid D (**4**)	K562	3.01	*Pseudoceratina purpurea*	South China Sea	Sesquiterpenoid	[7]
**43**	K562	4	*Luffariella variabilis*	South China Sea	Sesterterpenoid	[17]
**44**	K562	3.5	*Luffariella variabilis*	South China Sea	Sesterterpenoid	[17]
**45**	K562	2.9	*Luffariella variabilis*	South China Sea	Sesterterpenoid	[17]
**46**	K562	3.7	*Luffariella variabilis*	South China Sea	Sesterterpenoid	[1]
**47**	K562	3.2	*Luffariella variabilis*	South China Sea	Sesterterpenoid	[17]
**48**	K562	4.4	*Luffariella variabilis*	South China Sea	Sesterterpenoid	[17]
**49** (S)	K562	4.5	*Luffariella variabilis*	South China Sea	Sesterterpenoid	[17]
**49** (R)	K562	3.9	*Luffariella variabilis*	South China Sea	Sesterterpenoid	[17]
**55**	K562	3.5	*Luffariella variabilis*	South China Sea	Sesterterpenoid	[17]
sarcotragusolide A (**70a**)	K562	4.38	*Sarcotragus* sp.	South China Sea	Sesterterpenoid	[14]
sarcotragusolide A (**70b**)	K562	2.91	*Sarcotragus* sp.	South China Sea	Sesterterpenoid	[14]
pseudoceranoid D (**4**)	H69AR	7.74	*Pseudoceratina purpurea*	South China Sea	Sesquiterpenoid	[7]
pseudoceranoid E (**5**)	H69AR	2.85	*Pseudoceratina purpurea*	South China Sea	Sesquiterpenoid	[7]
**43**	H69AR	6.1	*Luffariella variabilis*	South China Sea	Sesterterpenoid	[17]
**44**	H69AR	5.2	*Luffariella variabilis*	South China Sea	Sesterterpenoid	[17]
**45**	H69AR	4.8	*Luffariella variabilis*	South China Sea	Sesterterpenoid	[17]
**46**	H69AR	4.9	*Luffariella variabilis*	South China Sea	Sesterterpenoid	[17]
**55**	H69AR	5.2	*Luffariella variabilis*	South China Sea	Sesterterpenoid	[17]
pseudoceranoid D (**4**)	MDA-MB-231	9.82	*Pseudoceratina purpurea*	South China Sea	Sesquiterpenoid	[7]
**48**	MDA-MB-231	4.3	*Luffariella variabilis*	South China Sea	Sesterterpenoid	[17]
enigmazole D (**206**)	MDA-MB-231	4.1	*Homophymia* sp.	Indonesia	Macrolide	[52]
phyllospongiane C (**82**)	MCF-7	1.1	*Phyllospongia foliascens*	South China Sea	Sesterterpenoid	[20]
phakefustatin A (**193**)	MCF-7	3.4	*Phakellia fusca*	South China Sea	Peptide	[48]
phyllospongiane C (**82**)	HT-29	1.2	*Phyllospongia foliascens*	South China Sea	Sesterterpenoid	[20]
enigmazole D (**206**)	HT-29	1.0	*Homophymia* sp.	Indonesia	Macrolide	[52]
phyllospongiane C (**82**)	NCI-H460	2.0	*Phyllospongia foliascens*	South China Sea	Sesterterpenoid	[20]
phakefustatin A (**193**)	NCI-H460	7.1	*Phakellia fusca*	South China Sea	Peptide	[48]
aaptolobamines A (**217**)	PC-3	3.4	*Aaptos lobata*	Australia	Polyamine	[56]
aaptolobamines B (**218**)	PC-3	4.1	*Aaptos lobata*	Australia	Polyamine	[56]
melophluosides A (**96**)	HeLa	9.7	*Melophlus sarasinorum*	Indonesia	Triterpenoid	[22]
phakefustatin A (**193**)	HeLa	6.2	*Phakellia fusca*	South China Sea	Peptide	[48]
enigmazole D (**206**)	A549	1.4	*Homophymia* sp.	Indonesia	Macrolide	[52]
enigmazole D (**206**)	PSN-1	1.1	*Homophymia* sp.	Indonesia	Macrolide	[52]
sarcotragusolide B (**71**)	AsPC-1	4.71	*Sarcotragus* sp.	South China Sea	Sesterterpenoid	[14]
phyllospongiane C (**82**)	C4-2-ENZ	0.7	*Phyllospongia foliascens*	South China Sea	Sesterterpenoid	[20]
tridiscorhabdin (**129**)	HCT-116	0.31	*Latrunculia biformis*	Antarctica	Alkaloid	[30]

K562 = leukemia cell line, H69AR = lung carcinoma cell line, MDA-MB-231 = breast cancer cell line, MCF-7 = breast adenocarcinoma cell line, HT-29 = colon cancer cell line, NCI-H460 = non-small cell lung cancer cell line, PC-3 = cancerous prostate cells, HeLa = immortal human cell line, A549 = lung adenocarcinoma cell line, PSN-1 = pancreatic adenocarcinoma cell line, AsPC-1 = pancreatic cancer cell line, C4-2-ENZ = enzalutamide-resistant prostate cancer cell line, HCT-116 = colon carcinoma cell line.

## Data Availability

No new data were created or analyzed in this study.
